# Revisiting socio-economic inequalities in sedentary leisure time in Sweden: An intersectional analysis of individual heterogeneity and discriminatory accuracy (AIHDA)

**DOI:** 10.1177/14034948221112465

**Published:** 2022-07-26

**Authors:** Lovisa Ericsson, Maria Wemrell, Martin Lindström, Raquel Perez-Vicente, Juan Merlo

**Affiliations:** 1 Unit for Social Epidemiology, Department of Clinical Sciences, Lund University, Sweden; 2 Department of Gender Studies, Lund University, Sweden; 3 Unit for Social Medicine and Health Policy, Department of Clinical Sciences, Lund University, Sweden; 4 Centre for Primary Health Care Research, Region Skåne, Sweden

**Keywords:** Health inequalities, physical activity, sedentarism, intersectionality, public health, Sweden

## Abstract

**Aims:**

Swedish public health reports have repeatedly provided information about socio-economic inequalities in sedentary leisure time, despite that, in the interest of health equity, physical activity should be equally distributed in the population. Such public health reports, however, neither consider the intersection of multiple socio-demographic factors nor the individual heterogeneity around group averages. Drawing on intersectionality theory, this study aimed to revisit previous findings on sedentary leisure time from Swedish public health surveys and demonstrate how the analysis of individual heterogeneity and discriminatory accuracy (AIHDA) can be used for analysing complex health inequalities.

**Methods:**

Using data from Swedish national public health surveys (2004–2015), we applied the AIHDA to define 72 intersectional groups by categories of age, gender, educational achievement, migration status and household composition. We then calculated (a) the absolute and relative risk of sedentary leisure time and (b) the discriminatory accuracy (DA) of the intersectional grouping.

**Results:**

The average risk of sedentary leisure time ranged from 5.8% among native-born, highly educated, young women living alone to 41.0% among immigrated young men, living alone, with low education. The risk was higher in strata comprising immigrated people with low education and lower in strata including native-born, highly educated people. However, the DA of the grouping was poor, indicating a substantial overlap of individual risk between groups.

**Conclusions:**

**Using the AIHDA and drawing on intersectionality, this study provides an improved mapping of the socio-economic distribution of sedentary leisure time in Sweden, with the poor DA suggesting universal rather than targeted physical activity interventions.**

## Introduction

The importance of physical activity for health and well-being is well known, and the World Health Organization [1] recommends that all adults undertake regular physical exercise. Meanwhile, in Sweden as elsewhere, a fundamental concern for public health policy is the prevention of unwarranted health inequalities [2]. The promotion of physical activity on equal terms is therefore an important public health issue [3].

Swedish public health reports have regularly documented socio-economic gradients in physical activity, showing, for instance, a higher prevalence of sedentary leisure time at lower levels of educational achievement [4,5]. Such reports are of clear relevance for measuring the prevalence of insufficient physical activity in the population, for identifying socio-economic inequalities and for guiding physical activity recommendations and interventions [6]. We argue that current public health reports could be improved, however, by providing (a) a more detailed, multi-categorical mapping of existing socio-economic inequalities and (b) information about the accuracy of such mapping for distinguishing between individuals who engage in sufficient physical activity and those who do not.

Public health reports on health inequalities have typically analysed socio-demographic variables as singular dimensions. This fails to capture the complex interplay between such dimensions, which can be identified by using an intersectional approach. Intersectionality theory builds on the core insight that social dimensions of differentiation, such as those of gender, ethnicity/racialisation and socio-economic position, are intertwined and should be analysed as such, as this is crucial for the understanding of the patterns of privilege and underprivilege that lead to unequal distribution of power and resources in society [7]. In quantitative research, a central aspect of considering intersectionality theory is the analysis of groups or strata defined by combinations of several social dimensions, with the aim of providing a more precise mapping of existing inequalities. The benefits of adopting an intersectional approach for illustrating complex health inequalities have been emphasised [8–11], and intersectionality is being increasingly applied in quantitative research on public health [12], including in studies of social disparities in physical activity [13–15].

Furthermore, public health reports typically apply a probabilistic, means-centric analytical approach that may provide insufficient information for understanding health inequalities. As noted elsewhere [10,16], the means-centric approach basically compares differences between group averages using measures such as relative risks or odds ratios. However, such comparison between group averages conveys several problems. First, categorising and comparing people on the basis of characteristics such as gender or immigration status may in itself sustain existing power structures through which groups are defined and oppressed (e.g. sexism, racism), and thereby maintain the very inequalities that are being analysed in the interest of their elimination. Second, if the distributions of individuals’ health values around group averages show considerable overlap between groups, targeted interventions to the most disadvantaged groups will be ineffective. In such cases, focusing only on groups with ‘unhealthy’ averages can lead to individuals with ill-health belonging to groups with ‘healthy’ averages being missed, and healthy individuals in the ‘unhealthy’ groups being treated unnecessarily. This situation relates to the problem of false-positives and false-negatives when using tests with low discriminatory accuracy (DA) [17], with the socio-economic grouping used to describe health inequalities here representing the test. Third, individuals belonging to groups with ‘unhealthy’ average values may become unnecessarily stigmatised, while there is a risk for false expectations among sick individuals who belong to ‘healthy groups’.

The analysis of individual heterogeneity and discriminatory accuracy (AIHDA) has been introduced as a suitable approach for analysing intersectional or complex health inequalities using either single- or multilevel regression (MAIHDA) [10,11,18]. Intersectional AIHDA provides a detailed, socio-economic mapping of the distribution of health in the population. It meanwhile considers the individual health heterogeneity around group averages by informing on the DA of the mapping. This information can be useful for the operationalisation of the concept of ‘proportionate universalism’ suggested by Marmot and Bell [19]. That is, public health interventions aiming to counter inequities in health ought to be universal but with a scale and intensity that is proportional to the level of need in specific population subgroups.

### Aim

Drawing on intersectionality theory, we aimed to apply the AIHDA to revisit previous findings from Swedish public health surveys demonstrating socio-economic inequalities in sedentary leisure time. We meanwhile aimed to demonstrate how the AIHDA can be used as an improved method for analysing complex inequalities in health.

## Methods

### Study population

In this cross-sectional, observational study, we used data from the Swedish National Public Health surveys (NPHS) provided to us by the Swedish Public Health Agency (PHA) after ethical approval. The survey covers health, lifestyle and living conditions and was conducted annually from 2004 to 2016 and biannually from 2018. Participants aged 16–84 years are randomly sampled individuals. The NPHS database contains updated individual and household information on socio-economic variables obtained by record linkage with population registers administrated by Statistics Sweden. The annual sample size has increased from 10,000 to 40,000 people. Response rates range from 60.8% in 2004 to 42.1% in 2018 [20]. For further information on sampling and response rates, we refer to Statistics Sweden [21].

**Figure 1.**
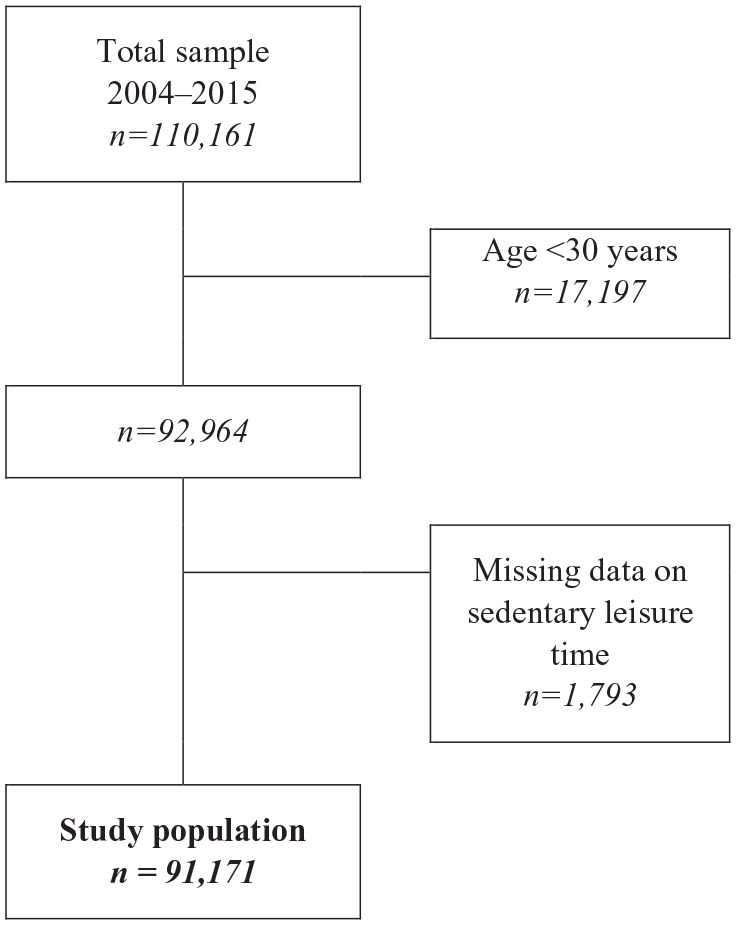
Flow chart describing the National Public Health Surveys study sample, as well as the inclusion and exclusion criteria and missing data.

Our sample consisted of pooled data from the 110,161 participants in the NPHS between 2004 and 2015. The question about physical activity was altered in 2016, and we therefore did not consider surveys from 2016 onwards. We excluded participants <30 years of age, since tertiary education was used as an indicator of socio-economic position, and those with missing data on education or sedentary leisure time. Thus, the final study population consisted of 86,793 individuals aged 30–84 years (Figure 1).

### Assessment of variables

#### Sedentary leisure time

Sedentary leisure time was defined by the response option ‘sedentary leisure time’ to the question ‘How much have you moved around and been physically active during the past 12 months?’. Respondents choosing any of the other three alternatives ‘moderate exercise in leisure time’, ‘moderate, regular exercise in leisure time’ or ‘regular exercise and training’ were assumed to have non-sedentary leisure time. The same definition has been used in reports published by the PHA [4,5], and the question has been validated [22].

#### Socio-economic and demographic variables

The independent variables were gender, educational achievement, migration status, age and household composition. Gender was self-reported and distinguished between men and women. Educational achievement was classified as pre high school, high school or post high school education, consistent with the categorisation used in earlier public health reports (translation from ‘gymnasium’ to high school by authors) [4,5]. Migration status was dichotomised as being born in Sweden (native) or outside of Sweden (immigrant). These variables were used as proxies for processes and inequalities associated with gender, class and racialisation [9,13–15]. Age was categorised into three groups (30–44, 45–64 and 65–84 years). Household composition distinguished between participants living alone or cohabiting. This was done due to an interest in the effects of age, such as unequal ageing [23], and marital/cohabitation status [24] on physical activity and thus on health.

#### Multi-categorical variable

**Figure 2.**
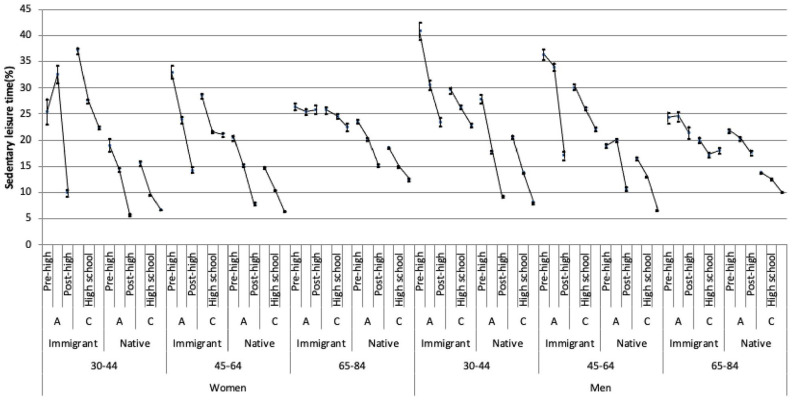
Prevalence of sedentary leisure time with 95% confidence intervals (vertical lines) for the 72 intersectional strata defined by combining the three categories of age, two categories of gender, three categories of educational achievement, two categories of migration status and two categories of household composition (A: living alone; C: cohabiting). The lines connecting the prevalence of sedentarism across groups with different levels of educational achievement show clear socio-economic gradients. The figures were obtained after weighting and imputation for missing values on educational achievement.

We created a multi-categorical variable with 72 strata by combining the socio-demographic variables (Figure 2). Native, highly educated, cohabiting men aged 30–44 years were used as the reference category in the analyses, as we assumed this group to have the highest structural privilege and thereby the most resources for a healthy (i.e. non-sedentary) lifestyle.

### Statistical analyses

We first described the temporal trends in sedentarism prevalence during 2004–2015 (Supplemental Material S1). We then pooled the data from 2004–2015 for the rest of our analyses, further describing sedentarism prevalence. Associations between the socio-demographic variables and the relative risk for sedentarism were quantified as prevalence ratios (PRs) with 95% confidence intervals (CIs) computed through Cox regression with a constant follow-up time equal to 1 [25]. We used six consecutive regression models. Model 1 included only age. The following models added, successively, gender (model 2), educational achievement (model 3), migration status (model 4) and household composition (model 5). The last model (Model 6) included the multi-categorical variable.

For each model, we calculated the area under the receiver operating characteristic curve (AUC). The AUC is constructed by plotting the true positive fraction (sensitivity) against the false-positive fraction (1-specificity) across thresholds of predicted probability of sedentarism and can be used as a measure of DA [17]. The DA can be defined, in this context, as the capacity of a model to discriminate between an individual with sedentary leisure time from an individual with non-sedentary behaviour. The DA can take a value between 0.5 and 1, with 1 representing perfect discrimination and 0.5 indicating no predictive accuracy. In this study, we classified the DA as ‘absent or very low’ (AUC=0.5–0.6), ‘poor’ (AUC >0.6–⩽0.7), ‘acceptable’ (AUC >0.7–⩽0.8), excellent (AUC >0.8–⩽0.9) or ‘outstanding’ (AUC <0.9–1) [26]. Lastly, we calculated the incremental change in AUC (ΔAUC) for each model compared to the previous one. Comparing the DA of model 6 to model 5, any statistical interaction of effects between the categories would be noted as an increase in the AUC.

The analyses were weighted using the sampling weights provided by Statistics Sweden. We performed multiple imputation by chained equations [27] for the missing data in the educational achievement variable using the Stata command ‘*mi*’.

IBM SPSS Statistics for Windows v25 (IBM Corp., Armonk, NY) and Stata v15 (StataCorp, College Station, TX) were used for the analyses.

### Ethical approval

This study was approved by the Swedish Ethical Review Authority (2019-01793) and by the PHA’s Ethical Council. NPHS respondents gave written informed consent prior to participation.

## Results

The average prevalence (i.e. absolute risk) of sedentary leisure time throughout the survey years was 14.4%, ranging from 13.4% in 2011 to 15.7% in 2015. A socio-economic gradient was identified, that is, the higher the education, the lower the risk (Table I). Further, the prevalence was higher among men, immigrants, those living alone and those aged 65–84 years than for women, natives, those cohabiting and those in younger age groups.

**Table I.** Prevalence of sedentary leisure time according to the Swedish National Public Health Surveys (NPHS) from 2014 to 2015 and by the included categories of age, gender, educational achievement, migration status and household composition.

	Sample		Population^a^	
	*n*/*N*	P (95% CI)	*n*/*N*	P (95% CI)
*Age (years)*				
30–44	2877/23,775	12.1 (11.7–12.6)	244,789/1,824,848	13.4 (13.4–13.5)
45–64	4754/38,792	12.3 (11.9–12.6)	324,898/2,356,251	13.8 (13.7–13.8)
65–84	4404/28,604	15.4 (15.0–15.8)	242,481/1,444,110	16.8 (16.7–16.9)
*Gender*				
Women	6283/49,269	12.8 (12.5–13.0)	395,245/2,821,718	14 (14–14)
Men	5752/41,902	13.7 (13.4–14.1)	416,920/2,806,491	14.9 (14.8–14.9)
*Educational achievement*				
Pre high school	2887/15,604	18.5 (17.9–19.1)	211,540/1,068,318	19.8 (19.7–19.9)
High school	5164/37,848	13.6 (13.3–14.0)	397,007/2,631,550	15.1 (15–15.1)
Post high school	2892/33,341	8.7 (8.4–9.0)	203,618/1,928,341	10.6 (10.5–10.6)
Missing	1092/4378	24.9 (23.7–26.2)	–	–
*Migration status*				
Immigrant	2579/11,212	23.0 (22.2–23.8)	229,293/932,675	24.6 (24.5–24.7)
Native	9456/79,959	11.8 (11.6–12.0)	582,872/4,695,534	12.4 (12.4–12.4)
*Household composition*				
Cohabiting	9039/74,053	12.2 (12.0–12.4)	609,406/4,528,074	13.5 (13.4–13.5)
Living alone	2996/17,118	11.0 (10.5–11.5)	202,759/1,100,135	18.4 (18.4–18.5)
*Survey year*				
2004	1327/9910	13.4 (12.7–14.1)	64,717/450,524	14.4 (14.3–14.5)
2005	658/4826	13.6 (12.7–14.6)	66,187/455,607	14.5 (14.4–14.6)
2006	659/4881	13.5 (12.5–14.5)	66,593/460,060	14.5 (14.4–14.6)
2007	618/4619	13.4 (12.4–14.4)	65,945/460,450	14.3 (14.2–14.4)
2008	1217/9095	13.4 (12.7–14.1)	67,613/461,677	14.6 (14.5–14.7)
2009	1088/8510	12.8 (12.1–13.5)	64,757/463,374	14.0 (13.9–14.1)
2010	1134/8345	13.6 (12.9–14.3)	69,906/470,378	14.9 (14.8–15.0)
2011	999/8175	12.2 (11.5–12.9)	63,189/472,909	13.4 (13.3–13.5)
2012	1028/8307	12.4 (11.7–13.1)	64,966/477,806	13.6 (13.5–13.7)
2013	1041/8167	12.7 (12.0–13.5)	69,202/482,844	14.3 (14.2–14.4)
2014	1083/8026	13.5 (12.7–14.2)	71,714/480,507	14.9 (14.8–15.0)
2015	1183/8310	14.2 (13.5–15.0)	77,376/492,073	15.7 (15.6–15.8)
*Total*	12,035/91,171	13.2 (13.2–13.2)	812,165/5,628,209	14.4 (14.4–14.5)

Note: The bottom row presents the average prevalence across the survey years. Values are number of individuals (*N*) with sedentary leisure time (*n*), and prevalence (P) of sedentarism in percentages with its 95% confidence interval (CI).

a Results obtained after weighting and imputation of missing values on educational achievement.

The multi-categorical analysis shows a heterogeneous distribution in the average risk of sedentary leisure time across the strata (Figure 2 and Table II). The prevalence of the reference group (i.e. native, cohabiting men aged 30–44 years with post high school education) was 8.1%. The highest prevalence was observed among immigrated men living alone, aged 30–44 years, with pre high school education (41.0%). Among women, the highest prevalence of 37.2% was found among immigrated, cohabiting people aged 30–44 years with pre high school education. The lines connecting the prevalence across groups with different levels of educational achievement (Figure 2) show clear socio-economic gradients.

**Table II.** Prevalence (P) as percentages and prevalence ratios (PR) of sedentary leisure time with 95% confidence intervals (CI) in the 10 multi-categorical strata with the lowest and the 10 intersectional strata with the highest prevalence and PR.

Age	Gender	Educational achievement	Migration status	Household composition	P (95% CI)	PR (95% CI)	*N*
*The 10 strata with the highest prevalence*							
30–44	Men	Pre high	Immigrant	Alone	41.0 (39.4–42.6)	5.09 (4.83–5.36)	3627
30–44	Women	Pre high	Immigrant	Cohabiting	37.2 (36.6–37.8)	4.62 (4.51–4.73)	26,103
45–64	Men	Pre high	Immigrant	Alone	36.4 (35.4–37.4)	4.52 (4.35–4.69)	8641
45–64	Men	High School	Immigrant	Alone	34.1 (33.3–34.8)	4.23 (4.1–4.36)	14,972
45–64	Women	Pre high	Immigrant	Alone	33.2 (32–34.4)	4.11 (3.93–4.31)	5933
30–44	Women	High School	Immigrant	Alone	32.6 (31–34.2)	4.05 (3.81–4.29)	3506
30–44	Men	High School	Immigrant	Alone	30.5 (29.6–31.5)	3.79 (3.65–3.94)	9391
45–64	Men	Pre high	Immigrant	Cohabiting	30.2 (29.7–30.8)	3.75 (3.66–3.85)	30,642
30–44	Men	Pre high	Immigrant	Cohabiting	29.6 (28.9–30.2)	3.67 (3.56–3.78)	17,580
45–64	Women	Pre high	Immigrant	Cohabiting	28.5 (28–28.9)	3.53 (3.45–3.62)	35,246
*The 10 strata with the lowest prevalence*							
65–84	Men	Post high	Native	Cohabiting	10.0 (9.9–10.2)	1.25 (1.22–1.28)	102,107
30–44	Women	Post high	Immigrant	Alone	10 (9.2–10.7)	1.24 (1.14–1.34)	6026
30–44	Women	High School	Native	Cohabiting	9.6 (9.5–9.7)	1.19 (1.17–1.21)	293,036
30–44	Men	Post high	Native	Alone	9.3 (9–9.5)	1.15 (1.11–1.18)	53,996
30–44	Men	Post high	Native	Cohabiting	8.1 (8–8.2)	Reference	273,963
45–64	Women	Post high	Native	Alone	7.9 (7.6–8.1)	0.98 (0.94–1.01)	54,721
30–44	Women	Post high	Native	Cohabiting	6.7 (6.6–6.8)	0.83 (0.82–0.85)	325,422
45–64	Men	Post high	Native	Cohabiting	6.6 (6.5–6.7)	0.82 (0.8–0.84)	251,017
45–64	Women	Post high	Native	Cohabiting	6.4 (6.3–6.5)	0.79 (0.78–0.81)	266,442
30–44	Women	Post high	Native	Alone	5.8 (5.5–6)	0.71 (0.68–0.75)	39,499

Note: The PR values have been calculated in the regression analysis for model 6 with native, cohabiting men aged 30–44 years with post high school educational achievement as the reference group. A complete list of all 72 intersectional strata from the Swedish National Public Health Surveys 2004–2015 can be found in Supplemental Material S2. The results were obtained after weighting and imputation for missing values on educational achievement.

Table III informs on the PRs obtained from the regression analyses. The average risk of sedentary leisure time was higher for men than women, for older than younger, for immigrants than for natives and for people living alone than for those cohabiting. The risk of sedentarism decreased as the level of educational achievement increased.

**Table III.** Prevalence ratios (PR) of sedentary leisure time with 95% confidence intervals (CI) obtained in five consecutive regression models using data from Swedish National Public Health Surveys 2004–2015.

	Model 1	Model 2	Model 3	Model 4	Model 5	Model 6^a^
	PR (95% CI)	PR (95% CI)	PR (95% CI)	PR (95% CI)	PR (95% CI)	
*Age*						
30–44	Reference	Reference	Reference	Reference	Reference	
45–64	1.03	1.03	0.95	0.97	0.96	
	(1.02–1.04)	(1.02–1.04)	(0.94–0.96)	(0.96–0.98)	(0.95–0.96)	
65–84	1.25	1.26	1.07	1.14	1.08	
	(1.25–1.26)	(1.25–1.26)	(1.07–1.08)	(1.13–1.14)	(1.07–1.08)	
*Gender*						
Men		Reference	Reference	Reference	Reference	
Women		0.94	0.96	0.94	0.93	
		(0.93–0.94)	(0.96–0.97)	(0.94–0.95)	(0.93–0.94)	
*Educational achievement*						
Pre high school			1.83	1.80	1.78	
			(1.79–1.86)	(1.76–1.83)	(1.75-1.82)	
High school			1.41	1.44	1.44	
			(1.38–1.44)	(1.41–1.48)	(1.41–1.47)	
Post high school			Reference	Reference	Reference	
*Migration status*						
Native				Reference	Reference	
Immigrant				2.01	2.02	
				(2.00–2.02)	(2.01–2.03)	
*Household composition*						
Cohabiting					Reference	
Living alone					1.32	
					(1.31–1.33)	
*Discriminatory accuracy (AUC)*						
AUC	0.527	0.537	0.551	0.596	0.601	0.612
∆AUC		0.010	0.014	0.045	0.005	0.011

Note: Included variables in each model are presented on the left. Also presented is the discriminatory accuracy of each model represented by AUC (area under the receiver operating characteristic curve) and ∆AUC, i.e., change in AUC compared to the previous model.

a Model 6 includes the same variables as model 5 but in the form of a multi-categorical variable with intersectional groups (i.e. strata) defined by the 72 combinations of the variables included in model 5 and using native men aged 30–44 with post high school educational achievement and cohabiting as the reference group. Information on the output of model 6 is found in Table 3 and Supplemental Material S2.

Table II presents the 10 strata with the highest and lowest absolute and relative risks for sedentary leisure time. The high-risk strata encompassed immigrated people with pre high school or high school education. Nine of the low-risk strata comprised people born in Sweden, and nine encompassed individuals with post high school education. More strata including men and individuals living alone were found among the high-risk strata and more women and cohabiting people in the low-risk ones. All age groups were found both amongst the low- and the high-risk strata, except for the oldest group which was not represented among the high-risk strata. Results on all 72 strata are found in Supplemental Material S2. A sensitivity analysis adjusting for survey year showed similar results (Supplemental Material S3).

The apparent heterogeneity in the strata distribution of average risk can be interpreted in light of the DA of the models (Table II). For model 1, the DA was ‘absent or very low’ (i.e. AUC=0.527). It gradually increased for each variable added, reaching the highest value in model 6 (AUC=0.612). In relative terms, the AUC increased with 16% from model 1 to model 6, but the DA obtained from the intersectional grouping was still ‘poor’. The ∆AUC of 0.011 between models 5 and 6 shows a very small interaction of effects between the variables defining the strata in the multi-categorical variable.

## Discussion

This study demonstrates how the AIHDA, drawing on intersectionality theory, can be used as an improved methodology for studying socio-economic inequalities in health. The AIHDA provided a detailed socio-economic mapping of the prevalence of sedentary leisure time in the adult, Swedish population. This mapping showed a heterogeneous distribution of sedentarism across 72 strata defined by age, gender, educational achievement, migration status and household composition. Such heterogeneity is often undetected in analyses focusing on singular social dimensions. We found conclusive differences in the average risk of sedentary leisure time, with strata including immigrated individuals with low educational achievement showing the highest risk, and strata encompassing native-born people with high educational achievement showing the lowest risk. Groups comprising different categories of gender, household composition and age were found among both high- and low-risk groups.

However, as expressed by the AUC values, the accuracy of the strata or variables for discriminating individuals with sedentary leisure time from those without sedentary leisure time was very low. The strata heterogeneity thus represented only a minor share of the total individual heterogeneity.

The information provided by public health reports is typically based on the comparison of differences between group averages. That approach may, as noted above, convey problems such as stigmatisation and sustainment of existing power inequalities, while potentially supporting false expectations and ineffective public health interventions resulting in over- or undertreatment. In addition to demonstrating a quantitative methodology drawing on intersectionality theory for the study of health inequalities, this study shows how the AIHDA integrates measures of DA to evaluate the relevance of the differences between group averages. In this way, the AIHDA aims to alleviate the problems created by an indiscriminate use of group averages [10].

In this study, the DA of the intersectional grouping was poor (0.612), indicating a substantial overlap of individual risk for sedentarism between groups. In accordance with the idea of proportionate universalism [19], this low DA does not support targeted interventions to the groups with the highest risk, but rather universal efforts aimed at the whole population. In general, the lower the DA, the more universal the intervention should be.

Our results on average risk differences are in line with previous research on physical activity, observing varying effects of income or education depending on sex/gender and ethnicity/racialisation [13–15]. These results, alongside ours, support the argument for an intersectional approach in the analysis of socio-economic inequalities in physical activity. Also in line with this, the PHA recently published a report on mental health based on NPHS data, adopting an intersectional perspective [28]. The intersectional analysis found larger inequalities than the conventional analysis, supporting the need for incorporating intersectional perspectives in public health reporting. We believe that the AIHDA approach can operationalise and further improve such analyses.

Despite the emerging support for the use of intersectional perspectives in health inequality research, consensus has not been reached on how to apply intersectionality in such study [9,12,29]. The approach outlined by Evans et al. [18] and by us [10] is based on (a) creating intersectional or multi-categorical strata from multiple socio-demographic categories and (b) not interpreting intersectional effects only based on statistical interaction of effects. We argue that multi-categorical analyses of health inequalities pointing to average risk differences between intersectional strata are relevant irrespective of whether statistical interactions of effects are present.

The intersectional strata used in this study could be improved through a more nuanced migration status variable and through the inclusion of additional dimensions, such as sexual orientation [13]. Our ability to do so was limited, however, as this would require a larger population sample. An additional limitation of this study lies in the self-reported nature of the dependent variable, and of gender, age and household composition. Moreover, the survey response rate was low, and higher among cohabiting persons with high income born in Sweden [30], which can affect the representativeness of the results. In addition, while we used the same definition of sedentary leisure time as that of the Swedish PHA, it should be noted that people who are more physically active at work may be less active during their leisure time. That pattern may be more common in groups with lower socio-economic position, and this study may therefore overestimate the socio-economic differences in physical activity overall. However, such overestimation is mitigated by, for example, exclusion from the work force due to sick leave or unemployment being more common in groups with lower educational attainment.

The AIHDA approach can be implemented using fixed effect [11] or multilevel random effect regression analyses (MLRA) [10]. MLRA provides conceptual and methodological advantages compared to fixed effects analyses [29]. Nevertheless, the fixed effect approach is more accessible and therefore suitable for public health reports, which is why we used it here.

## Conclusions

Applying the AIHDA and drawing on intersectionality theory, we obtained an improved mapping of the socio-economic distribution of sedentary leisure time in the adult population of Sweden. The DA of the intersectional grouping was poor, however, which suggests that public health interventions aiming to reduce sedentarism should be universal rather than targeted to specific population groups. AIHDA is an easily accessible method for examining complex socio-economic inequalities in health, which may lead to more effective public health interventions. However, to develop the intersectional analysis and applying MAIHDA, larger numbers of survey participants are needed. We argue that a larger NPHS sample and the use of intersectional AIHDA would provide a suitable framework for public health reporting on complex health inequalities in Sweden.

## Supplemental Material

sj-docx-1-sjp-10.1177_14034948221112465 – Supplemental material for Revisiting socio-economic inequalities in sedentary leisure time in Sweden: An intersectional analysis of individual heterogeneity and discriminatory accuracy (AIHDA) Click here for additional data file. Supplemental material, sj-docx-1-sjp-10.1177_14034948221112465 for Revisiting socio-economic inequalities in sedentary leisure time in Sweden: An intersectional analysis of individual heterogeneity and discriminatory accuracy (AIHDA) by Lovisa Ericsson, Maria Wemrell, Martin Lindström, Raquel Perez-Vicente and Juan Merlo in Scandinavian Journal of Public Health

sj-docx-2-sjp-10.1177_14034948221112465 – Supplemental material for Revisiting socio-economic inequalities in sedentary leisure time in Sweden: An intersectional analysis of individual heterogeneity and discriminatory accuracy (AIHDA) Click here for additional data file. Supplemental material, sj-docx-2-sjp-10.1177_14034948221112465 for Revisiting socio-economic inequalities in sedentary leisure time in Sweden: An intersectional analysis of individual heterogeneity and discriminatory accuracy (AIHDA) by Lovisa Ericsson, Maria Wemrell, Martin Lindström, Raquel Perez-Vicente and Juan Merlo in Scandinavian Journal of Public Health

sj-docx-3-sjp-10.1177_14034948221112465 – Supplemental material for Revisiting socio-economic inequalities in sedentary leisure time in Sweden: An intersectional analysis of individual heterogeneity and discriminatory accuracy (AIHDA) Click here for additional data file. Supplemental material, sj-docx-3-sjp-10.1177_14034948221112465 for Revisiting socio-economic inequalities in sedentary leisure time in Sweden: An intersectional analysis of individual heterogeneity and discriminatory accuracy (AIHDA) by Lovisa Ericsson, Maria Wemrell, Martin Lindström, Raquel Perez-Vicente and Juan Merlo in Scandinavian Journal of Public Health
