# Supplementary material for: Revisiting socio-economic inequalities in sedentary leisure time in Sweden: An intersectional analysis of individual heterogeneity and discriminatory accuracy (AIHDA)
Source: Scand J Public Health. 2022 Jul 26;51(4):570–8. doi: 10.1177/14034948221112465 (PMC10265284; doi:10.1177/14034948221112465)

# Supplementary material 1 (S1) for “Revisiting socioeconomic inequalities in sedentary leisure time in Sweden – an intersectional analysis of individual heterogeneity and discriminatory accuracy (AIHDA)”

***Supplementary material 1*** *(S1) for “Revisiting socioeconomic inequalities in sedentary leisure time in Sweden – an intersectional analysis of individual heterogeneity and discriminatory accuracy (AIHDA)” by Ericsson et al. in Scandinavian Journal of Public health. Temporal trends of sedentary leisure time (i.e., sedentary behavior) in the Swedish National Public Health Surveys (2004–2015) by the included variables age, gender, educational achievement, migration status and household composition. The figures are obtained after weighting and imputation for missing values on educational achievement.*


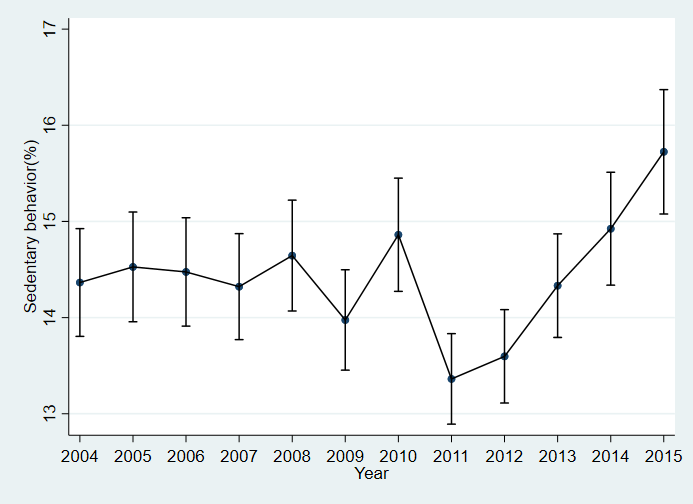

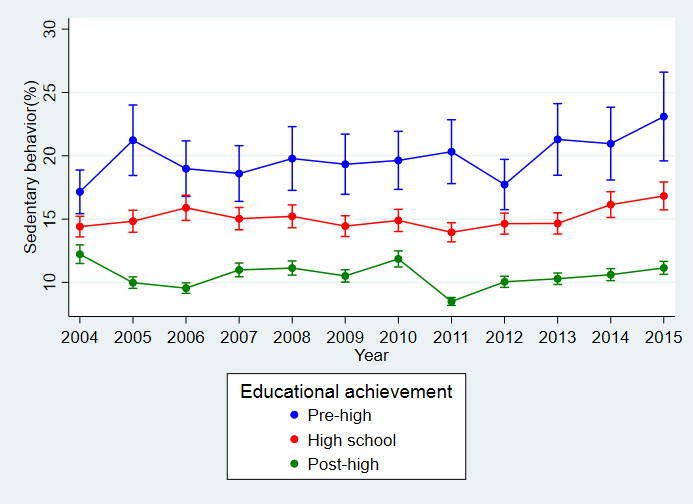

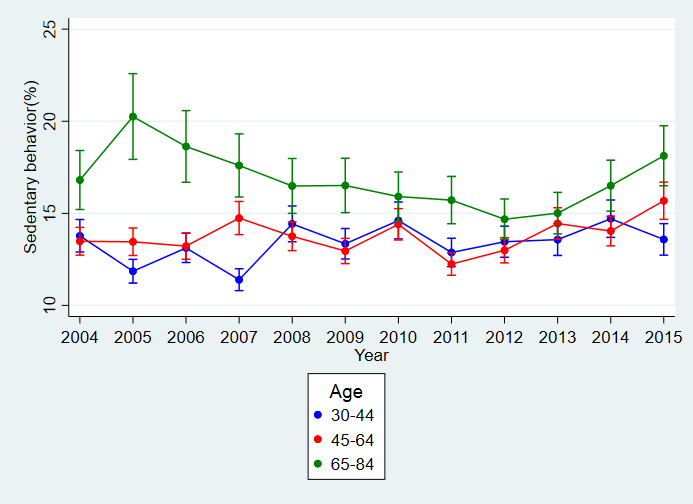

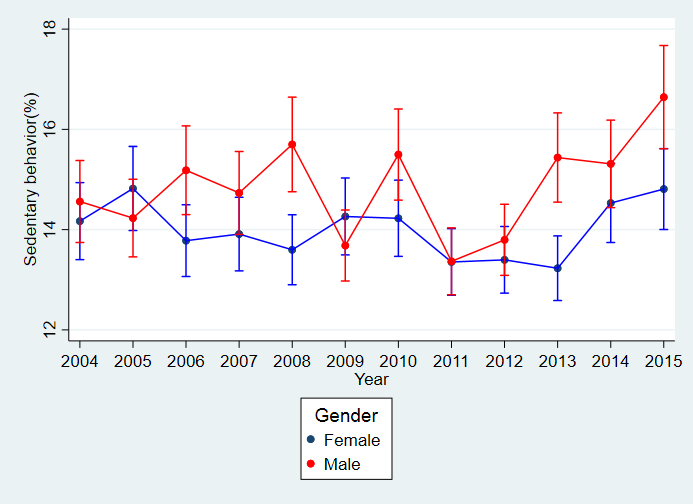

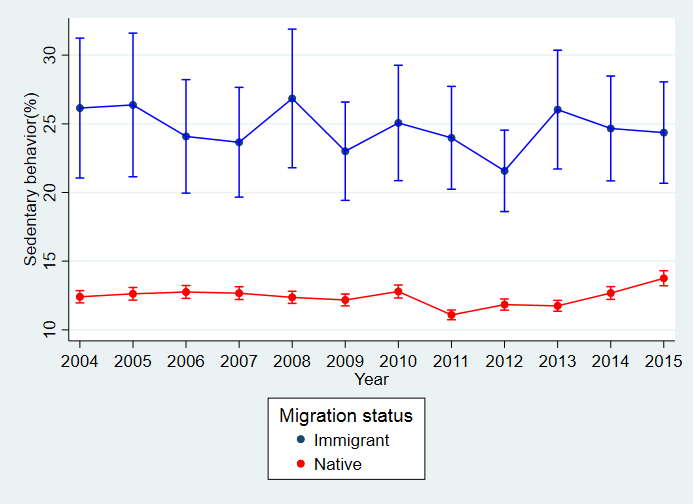

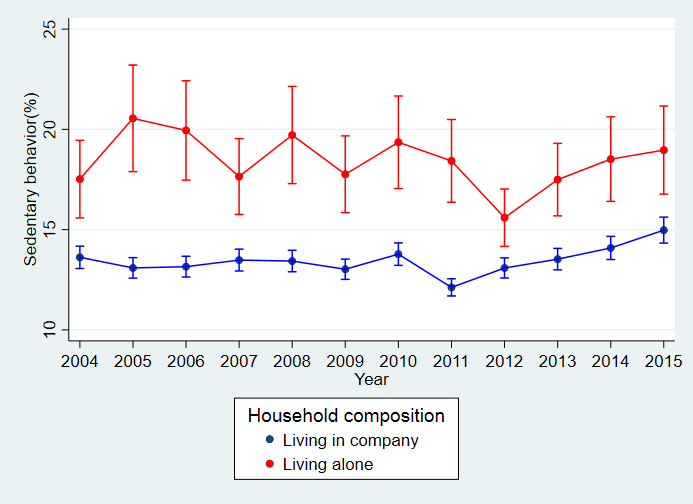

Supplement: sj-docx-1-sjp-10.1177_14034948221112465 – Supplemental material for Revisiting socio-economic inequalities in sedentary leisure time in Sweden: An intersectional analysis of individual heterogeneity and discriminatory accuracy (AIHDA) [file sj-docx-1-sjp-10.1177_14034948221112465.docx]
