# Supplementary material for: Revisiting socio-economic inequalities in sedentary leisure time in Sweden: An intersectional analysis of individual heterogeneity and discriminatory accuracy (AIHDA)
Source: Scand J Public Health. 2022 Jul 26;51(4):570–8. doi: 10.1177/14034948221112465 (PMC10265284; doi:10.1177/14034948221112465)
Supplement: sj-docx-2-sjp-10.1177_14034948221112465 – Supplemental material for Revisiting socio-economic inequalities in sedentary leisure time in Sweden: An intersectional analysis of individual heterogeneity and discriminatory accuracy (AIHDA) [file sj-docx-2-sjp-10.1177_14034948221112465.docx]

# Supplementary material 2 (S2) for “Revisiting socioeconomic inequalities in sedentary leisure time in Sweden - an intersectional analysis of individual heterogeneity and discriminatory accuracy (AIHDA)”

| ***Supplementary material 2*** *(S2) for “Revisiting socioeconomic inequalities in sedentary leisure time in Sweden - an intersectional analysis of individual heterogeneity and discriminatory accuracy (AIHDA)” by Ericsson et al. in Scandinavian Journal of Public health. Complete list of results pertaining to the 72 intersectional strata, from the Swedish National Public Health Surveys 2004–2015. Values are number of individuals in each stratum (N) with sedentary leisure time (n), prevalence (P) in percentages and prevalence ratios (PR) of sedentarism. The PR values have been calculated in the regression analysis for model 6. The 95% confidence intervals (CI) for P and PR are presented in parentheses. Prevalence and PRs are ordered from the lowest to the highest values. The figures are obtained after weighting and imputation for missing values on educational achievement.* | | | | | | | | |
| --- | --- | --- | --- | --- | --- | --- | --- | --- |
| *Age* | *Gender* | *Educational achievement* | *Migration status* | *Household composition* | *n* | *N* | *P  (CI 95%)* | *PR  (CI 95%)* |
| 30–44 | Women | Post-high | Native | Alone | 2273 | 39,499 | 5.80 (5.5–6) | 0.71  (0.68–0.75) |
| 45–64 | Women | Post-high | Native | Co-habiting | 17,039 | 266,442 | 6.40 (6.3–6.5) | 0.79  (0.78–0.81) |
| 45–64 | Men | Post-high | Native | Co-habiting | 16,579 | 251,017 | 6.60 (6.5–6.7) | 0.82  (0.8–0.84) |
| 30–44 | Women | Post-high | Native | Co-habiting | 21,878 | 325,422 | 6.70  (6.6–6.8) | 0.83  (0.82–0.85) |
| 45–64 | Women | Post-high | Native | Alone | 4305 | 54,721 | 7.90  (7.6–8.1) | 0.98  (0.94–1.01) |
| 30–44 | Men | Post-high | Native | Co-habiting | 22,076 | 273,963 | 8.01 (8–8.2) | Reference |
| 30–44 | Men | Post-high | Native | Alone | 5000 | 53,996 | 9.30 (9–9.5) | 1.15  (1.11–1.18) |
| 30–44 | Women | High School | Native | Co-habiting | 28,022 | 293,036 | 9.60  (9.5–9.7) | 1.19  (1.17–1.21) |
| 30–44 | Women | Post-high | Immigrant | Alone | 601 | 6026 | 10.00 (9.2–10.7) | 1.24  (1.14–1.34) |
| 65–84 | Men | Post-high | Native | Co-habiting | 10,257 | 102,107 | 10.00 (9.9–10.2) | 1.25  (1.22–1.28) |
| 45–64 | Women | High School | Native | Co-habiting | 43,631 | 416,034 | 10.50 (10.4–10.6) | 1.30  (1.28–1.32) |
| 45–64 | Men | Post-high | Native | Alone | 4008 | 37,472 | 10.70 (10.4–11) | 1.33  (1.28–1.37) |
| 65–84 | Men | High School | Native | Co-habiting | 24,595 | 195,891 | 12.60 (12.4–12.7) | 1.56  (1.53–1.59) |
| 65–84 | Women | Post-high | Native | Co-habiting | 10,878 | 86,558 | 12.60 (12.3–12.8) | 1.56  (1.52–1.6) |
| 45–64 | Men | High School | Native | Co-habiting | 53,595 | 410,880 | 13.00 (12.9–13.1) | 1.62  (1.59–1.64) |
| 30–44 | Men | High School | Native | Co-habiting | 44,362 | 322,342 | 13.80 (13.6–13.9) | 1.71  (1.68–1.74) |
| 65–84 | Men | Pre-high | Native | Co-habiting | 23,388 | 169,755 | 13.80 (13.6–13.9) | 1.71  (1.68–1.74) |
| 45–64 | Women | Post-high | Immigrant | Alone | 1726 | 12,000 | 14.40 (13.8–15) | 1.78  (1.7–1.87) |
| 30–44 | Women | High School | Native | Alone | 3818 | 26,439 | 14.40 (14–14.9) | 1.79  (1.73–1.85) |
| 45–64 | Women | Pre-high | Native | Co-habiting | 15,601 | 105,383 | 14.80 (14.6–15) | 1.84  (1.8–1.88) |
| 65–84 | Women | High School | Native | Co-habiting | 25,704 | 171,959 | 14.90 (14.8–15.1) | 1.86  (1.82–1.89) |
| 45–64 | Women | High School | Native | Alone | 13,766 | 90,919 | 15.10 (14.9–15.4) | 1.88  (1.84–1.92) |
| 65–84 | Women | Post-high | Native | Alone | 9338 | 61,174 | 15.30 (15–15.5) | 1.89  (1.85–1.94) |
| 30–44 | Women | Pre-high | Native | Co-habiting | 4343 | 27,810 | 15.60 (15.2–16) | 1.94  (1.88–2) |
| 45–64 | Men | Pre-high | Native | Co-habiting | 27,754 | 167,283 | 16.60 (16.4–16.8) | 2.06  (2.02–2.1) |
| 45–64 | Men | Post-high | Immigrant | Alone | 1457 | 8448 | 17.20 (16.4–18.1) | 2.14  (2.03–2.26) |
| 65–84 | Men | High School | Immigrant | Co-habiting | 5248 | 30,291 | 17.30 (16.9–17.8) | 2.15  (2.09–2.22) |
| 65–84 | Men | Post-high | Native | Alone | 4494 | 25,217 | 17.80 (17.3–18.3) | 2.21  (2.14–2.28) |
| 30–44 | Men | High School | Native | Alone | 11363 | 63,582 | 17.90 (17.6–18.2) | 2.22  (2.17–2.27) |
| 65–84 | Men | Post-high | Immigrant | Co-habiting | 3247 | 17,885 | 18.20 (17.6–18.7) | 2.25  (2.17–2.34) |
| 65–84 | Women | Pre-high | Native | Co-habiting | 25,433 | 136,963 | 18.60 (18.4–18.8) | 2.30  (2.26–2.35) |
| 45–64 | Men | Pre-high | Native | Alone | 8337 | 43,800 | 19.00 (18.7–19.4) | 2.36  (2.3–2.42) |
| 30–44 | Women | Pre-high | Native | Alone | 844 | 4422 | 19.10 (17.9–20.2) | 2.37  (2.21–2.54) |
| 65–84 | Men | Pre-high | Immigrant | Co-habiting | 4218 | 21,036 | 20.10 (19.5–20.6) | 2.49  (2.41–2.57) |
| 45–64 | Men | High School | Native | Alone | 18,004 | 89,562 | 20.10 (19.8–20.4) | 2.49  (2.45–2.54) |
| 65–84 | Women | High School | Native | Alone | 22,317 | 110,354 | 20.20 (20–20.5) | 2.51  (2.46–2.56) |
| 65–84 | Men | High School | Native | Alone | 10,568 | 52,011 | 20.30 (20–20.7) | 2.52  (2.46–2.58) |
| 45–64 | Women | Pre-high | Native | Alone | 5468 | 26,651 | 20.50 (20–21) | 2.55  (2.47–2.62) |
| 30–44 | Men | Pre-high | Native | Co-habiting | 9022 | 43,792 | 20.60 (20.2–21) | 2.56  (2.49–2.62) |
| 45–64 | Women | Post-high | Immigrant | Co-habiting | 13,050 | 61,759 | 21.10 (20.8–21.5) | 2.62  (2.57–2.68) |
| 65–84 | Men | Post-high | Immigrant | Alone | 962 | 4489 | 21.40 (20.2–22.6) | 2.66  (2.49–2.84) |
| 45–64 | Women | High School | Immigrant | Co-habiting | 15,834 | 73,283 | 21.60 (21.3–21.9) | 2.68  (2.63–2.74) |
| 65–84 | Men | Pre-high | Native | Alone | 10,552 | 48,577 | 21.70 (21.4–22.1) | 2.70  (2.63–2.76) |
| 45–64 | Men | Post-high | Immigrant | Co-habiting | 12,722 | 57,536 | 22.10 (21.8–22.5) | 2.74  (2.68–2.8) |
| 30–44 | Women | Post-high | Immigrant | Co-habiting | 18,819 | 84,027 | 22.40 (22.1–22.7) | 2.78  (2.73–2.83) |
| 65–84 | Women | Post-high | Immigrant | Co-habiting | 3193 | 14,205 | 22.50 (21.8–23.2) | 2.79  (2.69–2.89) |
| 30–44 | Men | Post-high | Immigrant | Co-habiting | 14,081 | 61,614 | 22.90 (22.5–23.2) | 2.84  (2.78–2.9) |
| 30–44 | Men | Post-high | Immigrant | Alone | 2550 | 10,828 | 23.60 (22.8–24.3) | 2.92  (2.81–3.04) |
| 65–84 | Women | Pre-high | Native | Alone | 20,529 | 87,148 | 23.60 (23.3–23.8) | 2.92  (2.87–2.98) |
| 45–64 | Women | High School | Immigrant | Alone | 4125 | 17,252 | 23.90 (23.3–24.5) | 2.97  (2.87–3.07) |
| 65–84 | Men | Pre-high | Immigrant | Alone | 1625 | 6662 | 24.40 (23.4–25.4) | 3.03  (2.88–3.18) |
| 65–84 | Women | High School | Immigrant | Co-habiting | 5974 | 24,255 | 24.60 (24.1–25.2) | 3.06  (2.97–3.15) |
| 65–84 | Men | High School | Immigrant | Alone | 2253 | 9137 | 24.70 (23.8–25.5) | 3.06  (2.93–3.2) |
| 65–84 | Women | High School | Immigrant | Alone | 5051 | 19,812 | 25.50 (24.9–26.1) | 3.16  (3.07–3.26) |
| 30–44 | Women | Pre-high | Immigrant | Alone | 312 | 1223 | 25.50 (23.1–28) | 3.17  (2.83–3.54) |
| 65–84 | Women | Pre-high | Immigrant | Co-habiting | 4986 | 19,333 | 25.80 (25.2–26.4) | 3.20  (3.1–3.3) |
| 65–84 | Women | Post-high | Immigrant | Alone | 3085 | 11,936 | 25.80 (25.1–26.6) | 3.21  (3.09–3.33) |
| 45–64 | Men | High School | Immigrant | Co-habiting | 18,387 | 70,375 | 26.10 (25.8–26.5) | 3.24  (3.18–3.31) |
| 30–44 | Men | High School | Immigrant | Co-habiting | 15,419 | 58,737 | 26.30 (25.9–26.6) | 3.26  (3.19–3.33) |
| 65–84 | Women | Pre-high | Immigrant | Alone | 4586 | 17,355 | 26.40 (25.8–27.1) | 3.28  (3.18–3.39) |
| 30–44 | Women | High School | Immigrant | Co-habiting | 15,860 | 57,530 | 27.60 (27.2–27.9) | 3.42  (3.35–3.49) |
| 30–44 | Men | Pre-high | Native | Alone | 3731 | 13,353 | 27.90 (27.2–28.7) | 3.47  (3.35–3.59) |
| 45–64 | Women | Pre-high | Immigrant | Co-habiting | 10,030 | 35,246 | 28.50 (28–28.9) | 3.53  (3.45–3.62) |
| 30–44 | Men | Pre-high | Immigrant | Co-habiting | 5198 | 17,580 | 29.60 (28.9–30.2) | 3.67  (3.56–3.78) |
| 45–64 | Men | Pre-high | Immigrant | Co-habiting | 9267 | 30,642 | 30.20 (29.7–30.8) | 3.75  (3.66–3.85) |
| 30–44 | Men | High School | Immigrant | Alone | 2868 | 9391 | 30.50 (29.6–31.5) | 3.79  (3.65–3.94) |
| 30–44 | Women | High School | Immigrant | Alone | 1143 | 3506 | 32.60 (31–34.2) | 4.05  (3.81–4.29) |
| 45–64 | Women | Pre-high | Immigrant | Alone | 1967 | 5933 | 33.20 (32–34.4) | 4.11  (3.93–4.31) |
| 45–64 | Men | High School | Immigrant | Alone | 5100 | 14,972 | 34.10 (33.3–34.8) | 4.23  (4.1–4.36) |
| 45–64 | Men | Pre-high | Immigrant | Alone | 3146 | 8641 | 36.40 (35.4–37.4) | 4.52  (4.35–4.69) |
| 30–44 | Women | Pre-high | Immigrant | Co-habiting | 9716 | 26,103 | 37.20 (36.6–37.8) | 4.62  (4.51–4.73) |
| 30–44 | Men | Pre-high | Immigrant | Alone | 1487 | 3627 | 41.00 (39.4–42.6) | 5.09  (4.83–5.36) |
