# Supplementary material for: Revisiting socio-economic inequalities in sedentary leisure time in Sweden: An intersectional analysis of individual heterogeneity and discriminatory accuracy (AIHDA)
Source: Scand J Public Health. 2022 Jul 26;51(4):570–8. doi: 10.1177/14034948221112465 (PMC10265284; doi:10.1177/14034948221112465)
Supplement: sj-docx-3-sjp-10.1177_14034948221112465 – Supplemental material for Revisiting socio-economic inequalities in sedentary leisure time in Sweden: An intersectional analysis of individual heterogeneity and discriminatory accuracy (AIHDA) [file sj-docx-3-sjp-10.1177_14034948221112465.docx]

# Supplementary material 3 (S3) for “Revisiting socioeconomic inequalities in sedentary leisure time in Sweden – an intersectional analysis of individual heterogeneity and discriminatory accuracy (AIHDA)”

| ***Supplementary material 3*** *(S3) for “Revisiting socioeconomic inequalities sedentary leisure time in Sweden – an intersectional analysis of individual heterogeneity and discriminatory accuracy (AIHDA)” by Ericsson et al. in Scandinavian Journal of Public health. Prevalence ratios (PR) of sedentarism when* ***adjusting for survey year*** *in the regression analysis for model 6, pertaining to the 72 intersectional strata from the Swedish National Public Health Surveys (2004–2015). PRs are presented from the highest to the lowest with 95% confidence intervals in parentheses. The figures are obtained after weighting and imputation for missing values on educational achievement.* | | | | | |
| --- | --- | --- | --- | --- | --- |
| *Age* | *Gender* | *Educational achievement* | *Migration status* | *Household composition* | *PR (CI 95%)* |
| 30–44 | Men | Post-high | Native | Co-habiting | Reference |
| 30–44 | Women | Post-high | Native | Alone | 0.73 (0.70–0.76) |
| 45–64 | Women | Post-high | Native | Co-habiting | 0.80 (0.78–0.81) |
| 45–64 | Men | Post-high | Native | Co-habiting | 0.83 (0.81–0.85) |
| 30–44 | Women | Post-high | Native | Co-habiting | 0.83 (0.82–0.85) |
| 45–64 | Women | Post-high | Native | Alone | 0.98 (0.95–1.01) |
| 30–44 | Men | Post-high | Native | Alone | 1.15 (1.11–1.18) |
| 30–44 | Women | High School | Native | Co-habiting | 1.19 (1.17–1.21) |
| 65–84 | Men | Post-high | Native | Co-habiting | 1.28 (1.25–1.31) |
| 30–44 | Women | Post-high | Immigrant | Alone | 1.30 (1.19–1.41) |
| 45–64 | Women | High School | Native | Co-habiting | 1.30 (1.28–1.32) |
| 45–64 | Men | Post-high | Native | Alone | 1.35 (1.31–1.40) |
| 65–84 | Men | High School | Native | Co-habiting | 1.52 (1.49–1.55) |
| 65–84 | Women | Post-high | Native | Co-habiting | 1.58 (1.54–1.61) |
| 45–64 | Men | High School | Native | Co-habiting | 1.62 (1.59–1.64) |
| 30–44 | Men | High School | Native | Alone | 1.72 (1.69–1.74) |
| 65–84 | Men | Pre-high | Native | Co-habiting | 1.74 (1.70–1.77) |
| 30–44 | Women | High School | Native | Alone | 1.79 (1.73–1.85) |
| 45–64 | Women | Post-high | Immigrant | Alone | 1.84 (1.75–1.93) |
| 45–64 | Women | Pre-high | Native | Co-habiting | 1.84 (1.80–1.87) |
| 65–84 | Women | High School | Native | Co-habiting | 1.85 (1.82–1.88) |
| 30–44 | Women | Pre-high | Immigrant | Alone | 1.89 (1.63–2.18) |
| 45–64 | Women | High School | Native | Alone | 1.89 (1.85–1.93) |
| 30–44 | Women | Pre-high | Native | Co-habiting | 1.96 (1.90–2.02) |
| 65–84 | Women | Post-high | Native | Alone | 1.97 (1.92–2.01) |
| 65–84 | Men | High School | Immigrant | Co-habiting | 2.02 (1.96–2.09) |
| 45–64 | Men | Pre-high | Native | Co-habiting | 2.06 (2.02–2.09) |
| 30–44 | Men | Post-high | Immigrant | Co-habiting | 2.23 (2.18–2.28) |
| 45–64 | Men | Post-high | Immigrant | Alone | 2.24 (2.13–2.36) |
| 30–44 | Women | Pre-high | Native | Alone | 2.26 (2.10–2.43) |
| 65–84 | Women | Pre-high | Native | Co-habiting | 2.31 (2.26–2.35) |
| 45–64 | Men | Pre-high | Native | Alone | 2.35 (2.29–2.41) |
| 65–84 | Men | Post-high | Native | Alone | 2.37 (2.29–2.44) |
| 65–84 | Men | Post-high | Immigrant | Co-habiting | 2.42 (2.33–2.50) |
| 65–84 | Women | High School | Native | Alone | 2.47 (2.42–2.52) |
| 65–84 | Men | High School | Native | Alone | 2.48 (2.42–2.54) |
| 45–64 | Men | High School | Native | Alone | 2.49 (2.44–2.54) |
| 65–84 | Men | Pre-high | Immigrant | Co-habiting | 2.5 (2.42–2.58) |
| 30–44 | Men | Pre-high | Native | Co-habiting | 2.5 (2.44–2.56) |
| 45–64 | Women | Pre-high | Native | Alone | 2.54 (2.46–2.61) |
| 65–84 | Women | High School | Immigrant | Co-habiting | 2.6 (2.52–2.68) |
| 45–64 | Women | High School | Immigrant | Co-habiting | 2.62 (2.56–2.67) |
| 65–84 | Men | Pre-high | Native | Alone | 2.66 (2.60–2.72) |
| 45–64 | Women | Post-high | Immigrant | Co-habiting | 2.67 (2.61–2.72) |
| 30–44 | Women | Post-high | Immigrant | Co-habiting | 2.7 (2.64–2.75) |
| 45–64 | Men | Post-high | Immigrant | Co-habiting | 2.76 (2.70–2.82) |
| 65–84 | Men | Post-high | Immigrant | Alone | 2.83 (2.67–3.00) |
| 30–44 | Men | Post-high | Immigrant | Alone | 2.84 (2.78–2.90) |
| 65–84 | Women | Post-high | Immigrant | Alone | 2.91 (2.79–3.02) |
| 30–44 | Men | Post-high | Native | Co-habiting | 2.93 (2.81–3.05) |
| 65–84 | Women | Pre-high | Native | Alone | 2.93 (2.87–2.98) |
| 65–84 | Women | High School | Immigrant | Alone | 2.96 (2.87–3.05) |
| 45–64 | Women | High School | Immigrant | Alone | 2.98 (2.88–3.08) |
| 65–84 | Men | High School | Immigrant | Alone | 2.99 (2.86–3.12) |
| 65–84 | Men | Pre-high | Immigrant | Alone | 3.08 (2.92–3.24) |
| 65–84 | Women | Post-high | Immigrant | Co-habiting | 3.18 (3.07–3.29) |
| 45–64 | Men | High School | Immigrant | Co-habiting | 3.24 (3.17–3.30) |
| 30–44 | Men | High School | Immigrant | Co-habiting | 3.24 (3.18–3.31) |
| 65–84 | Women | Pre-high | Immigrant | Co-habiting | 3.46 (3.36–3.56) |
| 30–44 | Women | High School | Immigrant | Co-habiting | 3.47 (3.40–3.54) |
| 30–44 | Men | Pre-high | Native | Alone | 3.51 (3.39–3.63) |
| 45–64 | Women | Pre-high | Immigrant | Co-habiting | 3.59 (3.51–3.68) |
| 30–44 | Men | Pre-high | Immigrant | Co-habiting | 3.62 (3.51–3.73) |
| 45–64 | Men | Pre-high | Immigrant | Co-habiting | 3.75 (3.66–3.84) |
| 30–44 | Men | High School | Immigrant | Alone | 3.8 (3.66–3.95) |
| 65–84 | Women | Pre-high | Immigrant | Alone | 3.81 (3.69–3.93) |
| 45–64 | Women | Pre-high | Immigrant | Alone | 4.03 (3.84–4.22) |
| 45–64 | Men | High School | Immigrant | Alone | 4.18 (4.06–4.31) |
| 30–44 | Women | High School | Immigrant | Alone | 4.19 (3.96–4.43) |
| 45–64 | Men | Pre-high | Immigrant | Alone | 4.58 (4.41–4.75) |
| 30–44 | Women | Pre-high | Immigrant | Co-habiting | 4.74 (4.63–4.85) |
| 30–44 | Men | Pre-high | Immigrant | Alone | 5.04 (4.79–5.32) |
